# Coerced to Restrain; Exploring the Association Between Institutional Pressure and Attitude Toward Restraints Among Trainees and Early Career Psychiatrists

**DOI:** 10.1192/j.eurpsy.2026.10979

**Published:** 2026-07-31

**Authors:** F. Monshizadeh Tehrani, S. M. Nouri, V. H. Santos, F. Mustač, L. Jishkariani

**Affiliations:** 1 Child and Adolescent Mental Health Center, Mental Health Services, Mental Health Services in the Capital Region of Denmark, Copenhagen, Denmark; 2 Zanjan University of Medical Science, Zanjan, Iran, Islamic Republic Of; 3 Department of Psychiatry and Mental Health, Cova da Beira Local Health Unit; 4 Faculty of Health Sciences, University of Beira Interior, Beira, Portugal; 5 Department of Psychiaty and Psychological Medicine, University Hospital Centre Zagreb, Zagreb, Croatia; 6 Department of psychiatry, Tbilisi state madical university, Tiblisi, Georgia

## Abstract

**Introduction:**

Restraint use in psychiatry is at the heart of ethical dilemmas between beneficence and non-maleficence. While sometimes necessary to prevent harm, it may violate autonomy, cause physical and psychological harm, and damage trust in mental health services.

Trainees and early-career psychiatrists (ECPs) are often required to prescribe restraints, sometimes even before feeling being adequately competent. Prescribing restraints may be experienced negatively and cause ethical conflicts, especially under institutional pressure (IP). Literature suggests that trainees and ECPs often hold less favourable attitudes toward coercive measures compared with other mental health staff groups. This discrepancy may potentially expose them to IP and moral distress or injury. Despite its importance, this area remains underexplored.

**Objectives:**

To assess the association between European trainees and ECPs’ attitudes toward restraints (ATR) and perceived adequacy of competence (PAC) with IP.

**Methods:**

A cross-sectional multinational study was designed by the Forensic Psychiatry Working Group of the European Federation of Psychiatric Trainees. The developed survey encompassed six scales, of which three were included in this study: (a) ATR (α = 0.805), (b) IP (α = 0.900) and (c) PAC (α = 0.933). The survey underwent face validation, expert review, clarity and fluency check, and a reliability test using Cronbach’s α to ensure internal consistency. Data were analysed by SPSS (V. 27). Normality was assessed by Shapiro–Wilk test and histograms. As normality assumptions were not met, associations were explored with Spearman’s correlations, and linear regression was used to examine whether PAC predicted IP. Statistical significance was set at p < 0.05.

**Results:**

Of the 206 participants from 24 European countries, 74% were trainees and 26% were ECPs (mean age = 30.5, SD = 5.4, range 24–56). The majority reported treating adult patients.

Analysis indicated a negative and significant correlation between both ATR (ρ = –0.26, p < .001) and PAC (ρ = –0.30, p < .001) with IP.

Furthermore, linear regression confirmed that PAC significantly predicts IP, F (1, 204) = 19.76, p < .001, explaining 8.8% of the variance (R^2^ = 0.088, adjusted R^2^ = 0.084). Higher PAC was associated with lower IP (B = –0.34, SE = 0.08, β = –0.30, t (204) = –4.45, p < .001).

**Conclusions:**

Our results suggest that trainees and ECPs with less favourable ATR and lower PAC may be exposed to greater IP, increasing the risk of moral injury. Based on our results PAC was found as a statistically significant predictor of IP, indicating that enhanced education and competence development may help reduce IP and protect trainees’ and ECPs’ mental health. However, as PAC accounted for only about 9% of the variance, other predictors may be involved, warranting further studies.

**Disclosure of Interest:**

None Declared

